# Systematic Approach to Mimic Phenolic Natural Polymers for Biofabrication

**DOI:** 10.3390/polym14071282

**Published:** 2022-03-22

**Authors:** Hyeju Han, Kyueui Lee

**Affiliations:** Department of Chemistry and Green-Nano Materials Research Center, Kyungpook National University, 80 Daehak-ro, Buk-gu, Daegu 41566, Korea; kd6886@knu.ac.kr

**Keywords:** polyphenols, biomimetics, biomaterials, biofabrication, biopolymers

## Abstract

In nature, phenolic biopolymers are utilized as functional tools and molecular crosslinkers to control the mechanical properties of biomaterials. Of particular interest are phenolic proteins/polysaccharides from living organisms, which are rich in catechol and/or gallol groups. Their strong underwater adhesion is attributed to the representative phenolic molecule, catechol, which stimulates intermolecular and intramolecular crosslinking induced by oxidative polymerization. Significant efforts have been made to understand the underlying chemistries, and researchers have developed functional biomaterials by mimicking the systems. Owing to their unique biocompatibility and ability to transform their mechanical properties, phenolic polymers have revolutionized biotechnologies. In this review, we highlight the bottom-up approaches for mimicking polyphenolic materials in nature and recent advances in related biomedical applications. We expect that this review will contribute to the rational design and synthesis of polyphenolic functional biomaterials and facilitate the production of related applications.

## 1. Introduction

Owing to a considerable increase in population with aging and industrial accidents, the demand for organ transplants has significantly increased [1]. Numerous individuals remain on waiting lists or pass away before transplantation because of the small number of organ transplant participants [2]. An alternative is to alter human-derived organs to artificial organs. Tissue engineering is the key technology used to develop artificial organs and has attracted significant attention; however, there are many hindrances to be resolved for the realization of artificial organs. Importantly, a strategy is required to build a three-dimensional (3D) structure from soft materials. Resultant materials should be either elastic or hard, but it is difficult to fabricate these shapes without heat or light for crosslinking.

Living organisms are not expected to directly utilize external stimuli but rather to adopt functional molecules for developing 3D structures. Phenolic molecules are typically utilized as key additives because of their unique abilities. Oxygen-responsive catechols and gallols are of particular interest since they can covalently crosslink nucleophilic molecules and can be crosslinked by themselves, resulting in mechanical transition. For example, the crosslinking mediated by catecholic molecules (3,4-dihydroxyphenylalanine) and histidine increased the Young’s modulus E of squid beak from approximately 0.05 to 5 GPa [3]. These functional groups exhibit strong binding affinity toward metallic ions (i.e., metal coordination), resulting in another type of crosslinked system [4].

Significant efforts have been undertaken to adopt and apply the underlying chemistries to develop 3D biomaterials for tissue engineering. These functional materials are called polyphenolic materials, where polyphenol refers to a material with many phenolic groups in its molecular structure. Catechol and gallol-functionalized polymers or synthetic polyphenols developed from phenolic building blocks are included in this category.

Herein, we provide guidelines for scientists and engineers to design and synthesize functional materials for 3D biofabrication using polyphenolic materials. First, we introduce fundamental studies on polyphenolic materials found in nature. Second, the synthetic approach for mimicking systems producing functional materials is discussed. Finally, applications of the developed materials for biomanufacturing are reviewed.

## 2. Mechanical Transformation in Biological Systems

Three major phenol derivatives are generally found in various biological tools; they are phenol, catechol, and gallol. The only difference is the number of hydroxyl groups on the benzoic site, but the kinetics significantly differ because of their molecular structure. These can solely work on the formation but occasionally play cooperative roles. Herein, we explain the difference between these chemicals resulting in different mechanical behaviors. Additives other than phenols (metallic ions and amines) or external stimuli (light) that induce mechanical enhancement are also discussed.

### 2.1. Phenolic Natural Polymers

Phenols found in nature mostly utilize tyrosine as a starting material, and tyrosine plays a vital role in the mechanical stabilization of structural tools in biological systems. Resilin and elastin, known as elastic and durable biomaterials, are typical examples. The elasticity originates from the dityrosine formation enabled by enzymatic oxidation [5]. The dityrosine chemistry is present in various living organisms, including dragonfly wings [5] and insect silk [6].

### 2.2. Catecholic Natural Polymers

Catechol is generally developed from the 3,4-dihydroxyphenylalanine (L-DOPA) structure originating from tyrosine. Tyrosinase oxidizes tyrosine to produce an additional hydroxyl group in the benzoic residue [7]. By following the additional enzymatic pathways, L-DOPA can be further transformed into dopamine and other neurotransmitters, such as norepinephrine [8,9]. These catecholic molecules are vital in the formation of functional biomaterials in living creatures. The most well-characterized material is an adhesive found in mussels. Mussel foot proteins contain over 20% of L-DOPA, which is unusual, compared with general proteins [10]. The strong adhesiveness is mainly attributed to catechols, which enable various chemical interactions, including hydrogen bonds, metal coordination, and pi-related bonds. Moreover, catechol is an oxygen-responsive functional group, which can induce oxidation, resulting in molecular crosslinking. This can mechanically harden the adhesive, which eventually stiffens, enabling mussels to strongly tether to neighboring substrates. Similar chemistry was found in insect cuticles. Insects are exposed to external threats from predators. To protect their body, insects develop mechanically strong cuticles as a protective armor. The cuticle generally comprises three chemical sources: polysaccharides (chitin), proteins, and catecholic molecules. Catecholic molecules including N-acetyldopamine (NADA) and N-β-alanyldopamine (NBAD) gradually crosslink the aforementioned biopolymers at the oxygen-abundant air/water interface [11]. This is called the tanning or sclerotization process. Occasionally, an amine-deprotected catecholic molecule is involved instead of NADA and NBAD. This accelerates the crosslinking kinetics, resulting in a mechanically strong tool. The grasshopper mandible is a particular example. Dopamine has functioned as a molecular crosslinker, enabling facile crosslinking [12]; the free amine plays a synergetic role in molecular crosslinking, resulting in mechanical reinforcement of grasshopper mandibles from approximately 3.8 to 6.1 GPa [12]. The synergetic role of amine is discussed further in Section 2.4.

### 2.3. Gallic Natural Polymers

Tunicates are surrounded by tough armor (tunic), which protects their inner organs from external forces. The toughness is attributed to crystalline cellulose nanofiber structures. Moreover, a peptide called tunichrome with gallol functionality (3,4,5-trihydroxyphenylalanine) plays a synergetic role in the formation of the tough tunic [13,14,15]. Gallols can contribute to the molecular crosslinking of tunic materials through metal coordination in which metals are provided from seawater [16]. Moreover, gallols can induce oxidative crosslinking between themselves and neighboring molecules (proteins). Owing to the aforementioned underlying chemistry found in tunicates, the tunicate can form a mechanically strong tunic, and the tunic is self-healing.

### 2.4. Synergetic Role of Amines and Metallic Ions

The function of the aforementioned biological tools originates from phenolic moieties, such as DOPA and TOPA; amines are frequently involved in material development. For example, various mussel foot proteins contain over 20% of lysine along with DOPA [17]. The primary amine group in lysine functioning as a nucleophile is probably grafted to the oxidized form of catechol (quinone), resulting in a C–N covalent bond formation, which stiffens the proteins. The elastic mussel foot proteins are eventually hardened, similar to concrete, so that the mussels can be strongly attached to rocks for their survival. Similar chemistry is found in other organisms. Tan et al. investigated the mechanical transition process in squid beaks [18]. Instead of lysine, histidine containing a secondary amine group was involved in the internal crosslinking of the squid beaks. Secondary amine is a strong nucleophile; therefore, it can be conjugated to the aromatic residue in DOPA, resulting in a His–DOPA crosslink. By controlling the concentration of amines, a squid can develop a mechanical gradient in its beak, which allows it to mechanically grab hard material using its soft tissue.

Metallic ions are often involved in the molecular crosslinking of phenolic materials through transition metal coordination. For example, zinc and copper have been utilized to reinforce the jaws of worm species [19]. Similarly, the mechanical properties of the spider fang and mussel byssus can be varied using metal–catechol complexes [20]. Likewise, tunicates with gallol moieties use metallic ions for metal complexation.

### 2.5. Natural Polyphenols

Phenolic, catecholic, and gallic molecules can be enzymatically oxidized in physiological systems, resulting in high molecular weight. Lignin biosynthesis is a typical example. Lignin can function as a molecular crosslinker between various types of polysaccharides, thereby controlling the rigidity of the plant components. In particular, hemicellulose–lignin crosslinking provides substantial stability to plants. The crosslinking is attributed to intermolecular and intramolecular hydrogen bonds [21] and originates from an enzymatically induced radical coupling between lignin and hemicellulose [22]. Oinonen et al. reported that hemicellulose in a tree can be covalently crosslinked with two or more lignins, forming a large covalent network [23]. Consequently, the mechanical property (tensile strength) of trees was negatively affected by the decomposition of lignin or hemicellulose [24].

## 3. Strategies to Design and Synthesize Polyphenolic Materials for Biofabrication

**Figure 1:**
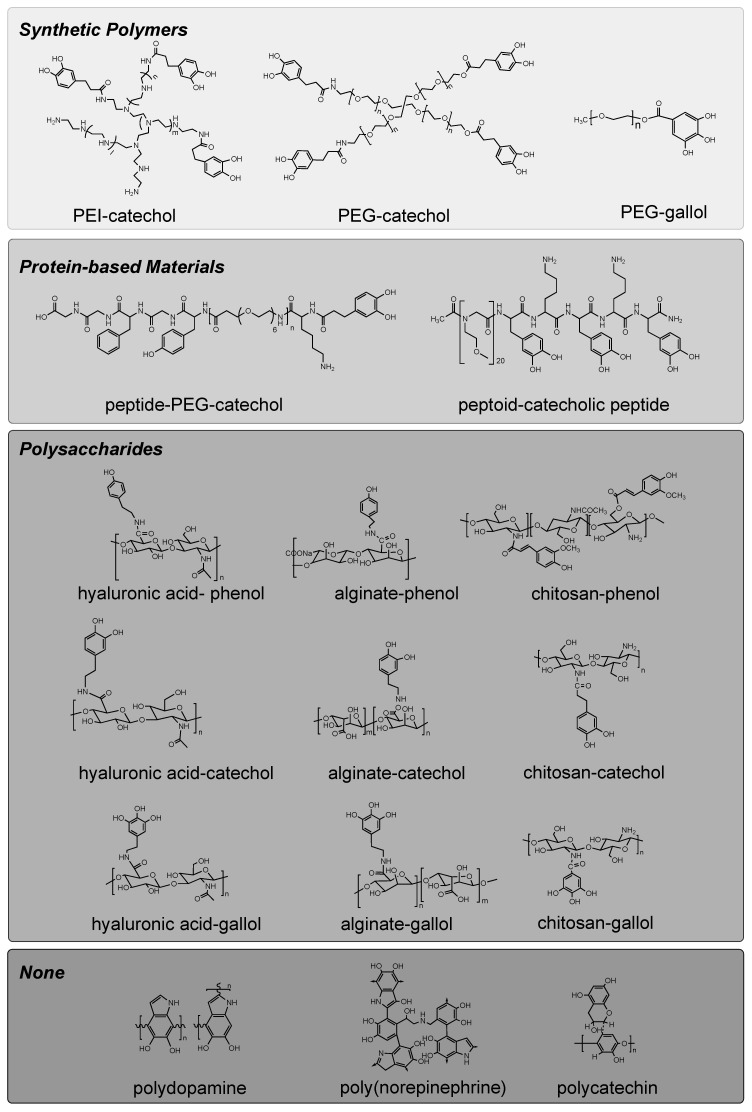
Schematic of representative polyphenolic materials.

Synthetic polyphenols allowing the mechanical transformation of biomaterials can be chemically designed by mimicking the aforementioned polyphenol chemistries in nature. Most reported approaches for material development used backbone resources. Herein, we introduce the representative backbone materials (Table 1, Figure 1) and relevant methods for grafting phenols on these materials, with a short guideline to select suitable polyphenolic materials in particular circumstances.

### 3.1. Choice of Backbone Materials

#### 3.1.1. Synthetic Polymers

Previously, phenolic groups were embedded in polymeric backbones, including polyethyleneimine [25,26], polyethylene glycol [67], polyethylene [29], polyester [30,31], epoxy [32,33], polypropylene [34,35], polytetrafluoroethylene [36,37], and polystyrene [38,39]. As a result, the original mechanical performance of the polymers significantly improved. Owing to phenolic groups, the modified polymers exhibited better molecular adhesion properties. The origin of the adhesion is attributed to chemical interactions between phenolic groups and the neighboring substrate, including various noncovalent interactions such as hydrogen bonds, electrostatic interactions, and pi-related bonds. Covalent interactions are often involved in the adhesion property when the underlying substrates are either metals or nucleophilic substances. Grafting of phenolic groups was performed noncovalently or covalently; this is further discussed in Section 3.2.

#### 3.1.2. Peptide-Based Materials

Protein derivatives or analogs are frequently used as backbone materials for polyphenolic materials. These include peptides [40,41], peptoids [42,43], extracellular matrix [44], mussel adhesive proteins [45,46], and polypeptides [47,48]. Consequently, the adhesiveness of the backbone materials increased. The chemical crosslinking induced using adhesive functional groups also stimulated mechanical hardening. For example, after the catechol conjugation on the peptides, the materials exhibited a strong binding affinity toward inorganic nanoparticles [40]. The impact of catechol moiety substitutions on the aromatic ring on adherence to the surface was confirmed using single-molecule force spectroscopy with atomic force microscopy (AFM) [41]. Similarly, peptoids that mimic the structure of peptides were adopted as another backbone material. The incorporated phenolic residues can be used as a molecular crosslinker, thereby enabling various conjugation chemistries. Boronate ester formation based on catechol–boronic acid interaction is a typical application. A dynamic covalent assembly was achieved through this chemistry, resulting in molecular ladder and grid structures [42]. Statz et al. reported a synthetic polypeptoid with biomimetic anchoring groups (catecholamines), which enabled robust water-resistant antifouling surfaces with long-term stability in the biological environment [43].

The oxidation of catechol-conjugated decellularized ECM resulted in crosslinking between catechols [44]. Therefore, the mechanical property of the decellularized tissue significantly increased. Owing to the increased adhesiveness with high mechanical stability, the shape of the materials could be controlled without any supporting material, which was difficult to achieve in the original decellularized tissue-based hydrogels.

There is a strategy for producing natural polyphenols by employing synthetic biology. For example, recombinant premature MAPs were produced using cDNA coding [45,46], accompanied by post-treatments of the recombinant proteins to insert additional hydroxyl groups. For example, tyrosine residues can be converted into DOPA by tyrosinase treatment [45,46,47]. The direct grafting of phenolic molecules is another option. In this regard, Lee et al. reported elastin-like polypeptides (ELP) where catechols were conjugated through 1-ethyl-3-(3-dimethylaminopropyl)carbodiimide/N-hydroxysuccinimide (EDC/NHS) chemistry by utilizing carboxylic groups on the polypeptides [48]. As a result, the stability of the resultant hydrogel highly increased, as confirmed by rheological studies.

#### 3.1.3. Polysaccharides

Polysaccharide-based backbone materials for producing polyphenolic materials include chitosan [49,50], hyaluronic acid (HA) [51,52,53], alginate [54,55], collagen [56], and cellulose [57]. The majority of the grafting methods rely on EDC/NHS chemistry. Three representative phenolic groups (phenol, catechol, and gallol-containing molecules) have been grafted to backbone materials using the aforementioned chemistry (Section 3.2). Even with one hydroxyl group difference, the kinetics for the crosslinking reactions significantly differ. Comparative studies have been performed to demonstrate these differences [52]. The results implied that the mechanical properties of polyphenolic materials can be controlled by chemical design. Gallol-conjugated materials typically exhibit high oxygen sensitivity, resulting in rapid oxidative crosslinking, compared with those of phenolic/catecholic materials, which are desired in the 3D construction of biomaterials [53]. Polyphenols exhibit strong adhesiveness because of the capability of various chemical interactions, such as electrostatic interactions, π–π stacking, and hydrogen bonding; additives (enzymatic or chemical oxidants) are often involved in facilitating the crosslinking reactions, which may affect the crosslinking behavior of polyphenolic materials, resulting in mechanical stabilization. Horseradish peroxidase, hydrogen peroxide solution, sodium periodate, ammonium persulfate, and iron (III) chloride are typical examples. As a result of addition, the materials exhibited better adhesion and cohesion; hence, the resultant materials exhibited better mechanical properties. For example, the elastic modulus of the catechol-conjugated chitosan can be spontaneously increased from 0 to 7.3 kPa in H_2_O_2_ with hematin [68].

#### 3.1.4. Polyphenols That Do Not Require a Backbone Material

Polyphenolic materials that spontaneously develop from a single phenolic monomer exist. The monomeric options that can be used to develop polyphenols are expanding. Theoretically, molecules with a functional group likely to be oxidized in a basic solution possess potential as phenolic building blocks. Here, we described a few representative phenolic monomers and their derivative polyphenols. These include polydopamine [58,59], polynorepinephrine [60,61], polytannic acid [62,63], pyrocatechol violet [64], and polycatechin [65,66].

Polydopamine is the most well-characterized and well-adopted synthetic polyphenol. Sequential oxidations of dopamine in an alkalic solution resulted in quinone and 3,4-dihydroxyindole (DHI) structures, which can be further crosslinked via covalent and noncovalent interactions. The procedures for polydopamine development are under ongoing investigation; however, it is accepted that the aforementioned key molecules play a vital role in the formation of polydopamine [58,59,69,70]. Similar chemistries are found in other synthetic polyphenols; however, the underlying crosslinking mechanisms are even more unclear compared with the polydopamine formation.

The advantage of this kind of polyphenolic materials is their material-independent coating ability. The polyphenolic coating layers can be introduced to virtually any kind of material using a simple one-step dip-coating process. This provides phenolic functionality to the nonfunctional materials. Owing to the presence of phenolic groups at the interface, polyphenol-coated materials exhibit molecular adhesiveness, which is unachievable in their original form. For example, a tannic acid-coated substrate showed strong binding affinity toward PEG [71] and cells [72], resulting in the antifouling surface and a circulating tumor cell-capturing platform, respectively. The polyphenolic composites generally exhibited strong mucoadhesion [73]. Thus, the materials were adapted, particularly to the biomaterial field.

Considering the strong binding affinity between phenolic groups, polyphenol-coated materials are likely to strongly bind with each other, resulting in mechanical reinforcements in several soft materials. Thus, polyphenol-coated composite materials commonly exhibit better mechanical and thermal stability compared with that of the original materials in ambient [74,75,76,77] or extreme environments [78].

#### 3.1.5. Additive Groups Other Than Phenols Supporting the Crosslinking Behavior

In polyphenol-based hydrogel systems, the degrees of crosslinking and adhesiveness are generally inversely proportional to each other since the origin of both abilities relies on phenolic moieties. To preserve catechol functionality during the crosslinking of hydrogels, aldehyde-modified polysaccharides are alternatively used to induce a Schiff base formation rather than oxidative polymerization [55].

Click chemistry, a copper-catalyzed azide–alkyne cycloaddition (CuAAC), was introduced to improve the cohesion and wet adhesion of mussel-inspired bioadhesives. The crosslinkers azide and alkyne were introduced in addition to the catechols, thereby enabling dual crosslinking systems and allowing catechol groups to participate in adhesion. Thus, the adhesion strength was further improved [79].

Metallic ions are often involved in the development of the polyphenolic materials starting from phenolic building blocks described in Section 3.1.4, which induces coordination-driven assembly facilitating the biofabrication of the polyphenolic materials [80,81].

### 3.2. Methodology for the Integration of Phenols to Backbone Materials

#### 3.2.1. EDC/NHS Chemistry

**Figure 2:**
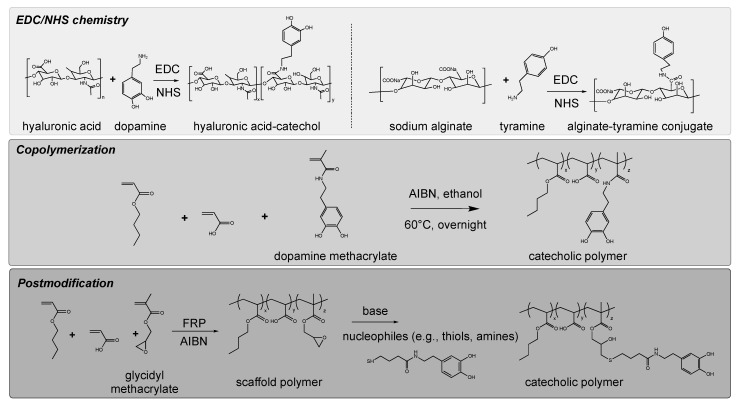
Synthetic scheme for producing polyphenolic materials.

EDC/NHS chemistry is the most well-adapted method for grafting phenolic groups to backbone materials. Polysaccharides are generally employed as backbone materials. In detail, either amine (chitosan/collagen) or carboxylic (HA/alginate) side chains on backbone materials are used as anchoring functional groups (Figure 2). The phenolic molecules for the EDC/NHS chemistry are chosen by considering the side-chain type. For example, a carboxyl group (–COOH) of polysaccharides was activated via EDC/NHS chemistry and became a conjugate by forming an amide bond with an amino group (–NH_2_) from dopamine hydrochloride [49,50,51,52,53,54,55,56,57,82]. In contrast, amine-rich chitosan backbone was reacted with hydrocaffeic acid with a carboxylic acid group for the catechol functionalization [49,50].

Certain backbones do not possess a functional group for conjugation chemistry. In this case, a pretreatment of the backbone materials is applied to the functionalization. For example, the carboxylate content in cellulose can be amplified through 2,2,6,6-tetramethylpiperidine-1-oxyl radical (TEMPO)-mediated oxidation [57]. This allows an amidation reaction (EDC/NHS chemistry) between TEMPO-oxidized cellulose and dopamine hydrochloride.

#### 3.2.2. Copolymerization

The phenolic functionality can be incorporated into polymeric materials by copolymerizing phenolic monomers (Figure 2). Allyl-, methacrylic-, and methacrylamide-phenols are generally utilized. For example, dopamine methacrylamide was copolymerized with common pressure-sensitive adhesive monomers (butyl acrylate and acrylic acid) to produce acrylic wet adhesives [83]. Similarly, dopamine methacrylamide was copolymerized with an allyl-containing monomer to functionalize an oil/water separation membrane [84].

#### 3.2.3. Post-Modification

The aforementioned copolymerization approach occasionally results in unexpected side reactions. For example, the high polydispersity of a resultant polymer was achieved because of the radical scavenging effect of the phenolic groups. Post-modification methods can be alternatives to minimize side effects. Conceptually, the scaffold polymer is initially synthesized, and phenolic molecules are subsequently introduced (Figure 2). For example, we previously fabricated a scaffold polymer comprising three different building blocks [32]. Two of them were conventional monomers for acrylic adhesives, and the third was the functional group (glycidyl methacrylate) with epoxide residue. Nucleophilic molecules can be easily grafted to this epoxide residue through a ring-opening reaction. This includes the conjugation of phenolic molecules. The advantages of this approach are as follows: (1) the molecular weight of the resultant polymers can be fixed, allowing fair comparison between the polymers, and the (2) exotic monomer preparation step can be skipped.

#### 3.2.4. Self-Oxidation

Self-oxidation is the process that does not require a backbone material during polyphenol synthesis. The representative polyphenol is the dopamine-derived polydopamine in which the catechol in the dopamine is oxidized in an alkaline tris buffer to form quinone. Subsequently, a nucleophilic reaction occurs with the primary amine to form a DHI structure. The covalent and noncovalent interactions of these intermediate products gradually form polydopamine [69]. Similar chemistry was found in polynorepinephrine synthesis [85]. The only difference is the molecular structure of the intermediates that is involved in the polyphenol synthesis. For example, in the oxidative polymerization of norepinephrine, 3,4-dihydroxybenzaldehyde is newly generated rather than DHI derivatives. The additional hydroxyl group allows a smoother coating on the target substrate, compared with that of polydopamine. Analogous phenolic building blocks such as L-DOPA also undergo a comparable oxidation chemistry, resulting in a molecular adhesive functional coating.

### 3.3. Selection Guideline

Polyphenolic materials have pros and cons depending on their backbone types. Based on previous reports, we propose a brief guideline for selecting backbone material that is suitable for targeted applications (Table 2). We suggest four key factors to be considered for polyphenolic material fabrication; they are biocompatibility, accessibility, degradation, and mechanical properties.

Owing to the simplicity of the chemical structures of synthetic polymers, fundamental studies on synthetic polymers are relatively easy to perform. The physicochemical properties of the materials can be controlled by altering the monomer type. Moreover, diverse monomer choices enable the production of mechanically strong materials (tough gels) that are hardly achievable using polyphenolic materials with different backbone systems. In contrast, biological responses from synthetic polymers are difficult to predict because of their unnatural structures. Thus, only a few polymers with biodegradable bonds (ester bonds) are approved for biological applications, which is the current limitation of this material.

Considering the nature of protein-based polyphenolic materials, they are highly biocompatible with enzymatic degradation ability. However, given that the material production is typically achieved using synthetic biological approaches, there are adverse consequences for scale-up processes.

As previously described, the majority of the previously reported polysaccharide-based polyphenolic materials can be prepared via simple one-step conjugation chemistry (EDC/NHS chemistry); therefore, they are highly accessible. Moreover, polysaccharides are abundant in nature, and they can be easily extracted from natural resources. Considering that several polysaccharides are components of the human body (e.g., HA), polysaccharide-based polyphenols typically exhibit high biocompatibility. However, there is a risk that allergic proteins can also be extracted during the extraction process for natural polysaccharides.

Polyphenolic materials that are developed from a basic building block (e.g., dopamine) are easy to synthesize. The synthesis procedures resemble those for melanin synthesis; therefore, the related materials including polydopamine generally exhibit high biocompatibility. However, the underlying chemistry is extremely complex. Moreover, the molecular weight range is broad and hard to define. These are the bottlenecks in the realization of the relevant materials.

## 4. Applications

Inspired by the underlying chemistries of functional natural polyphenols, relevant synthetic polyphenols have been chemically designed and utilized for numerous biomaterials. Various applications have been suggested from a decade of research. Herein, we present representative applications of polyphenolic biomaterials with a focus on biomanufacturing tools.

### 4.1. Biomedical Adhesives

Bioadhesives are an example of polyphenol-based applications, which are fundamentally studied and realized for practical use [86]. The strong tissue adhesion between bioinspired adhesives and tissue is attributed to the nucleophilic addition of amine/thiol groups from amino acids on adhesives [87,88]. Here, a catechol or gallol group that can be spontaneously rearranged into a quinone reacts as an electrophile.

Adhesives have been developed using biological and chemical approaches. For example, Cha et al. have developed biologically synthesized mussel adhesive proteins as a biomedical tissue adhesive [89,90]. Lee et al. developed chitosan-based tissue adhesives [86,91]. Owing to their immediate crosslinking behavior, they can be used as surgical tissue adhesives and for medical sealants and hemostats that can function as alternatives for surgical sutures. The alternation in the backbone structures or combination of additional crosslinkers often provides extra functionalities in bioadhesives. For example, the catechol-functionalized poly(y-glutamic acid) exhibited strong adhesion and tissue-like elastomeric mechanical properties with high biocompatibility, stability, and biodegradability [92]. In particular, the decomposition rate can simply be adjusted by controlling the degree of crosslinking. Ryu et al. reported the synergetic effect of a thiolated pluronic polymer in a polyphenolic hemostat due to the acceleration of the crosslinking [93]. External stimulation can improve the hemostatic ability of bioadhesives. For example, the photo-oxidation of tyrosine facilitates the crosslinking of dityrosine, resulting in the rapid closure of a bleeding site [94]. 

### 4.2. Functional Biocoatings

The interfacial engineering of biomedical implants is essential for improving biocompatibility, and the secondary modification of implant materials expands the applicability of these materials. Polydopamine and other polyphenolic multifunctional coatings have been adopted as tools for the interfacial engineering of biomaterials, and this can be realized by their inherent molecular adhesive properties. The coating materials exhibit high biocompatibility and possess the potential to acquire other abilities, such as osteoconductivity [95,96], antifouling ability [97,98], and acceleration of cell proliferation [98,99], after integration with the appropriate biomolecules.

### 4.3. Bioprinting

The polyphenolic materials mimicking the biological system in nature are ideal for bioinks. In particular, the phenolic crosslinking that induces the mechanical transition of biomaterials plays a key role in biofabrication. For example, catechol-conjugated chitosan can solely build a 3D structure in the serum media without the aid of external stimulation or crosslinking agents [50]. Bioprinting can be achieved by catechol-mediated intermolecular and intramolecular interactions between serum proteins. The addition of vanadyl ion even enhanced the mechanical properties of the 3D-printed biomaterials owing to the extra crosslinking through catechol–metal complexation. 

Likewise, gallol-functionalized polyphenolic materials (e.g., hyaluronic acid-gallol) are oxidatively crosslinked in the ambient condition, resulting in a 3D construction [100]. The distinct feature of gallic materials is that a dynamic hydrogen bond is immediately generated owing to the abundant hydroxyl groups, providing shear-thinning ability. Thus, the gallol-based hydrogel ink could be applied to an extrusion-based 3D printer for 3D printing [100]. Moreover, the phenolic groups can mechanically stabilize a 3D-printed structure. For example, the catechol-tethered alginate hydrogel showed better stability in the physiological condition (i.e., wet environment) compared with the alginate-based hydrogel. Improvement is attributed to internal crosslinking between catechol groups [101].

The advantage of the phenolic functionality does not end with mechanical stabilization; it can further be expanded by applying their phenolic functionality. For example, a conductive polyphenol-based hydrogel can be developed using the redox chemistry, in which silver nanoparticles can be spontaneously formed on the hydrogel via gallol redox chemistry [102]. The conductivity of the polyphenolic hydrogels can be easily controlled by mixing conductive and nonconductive microgels during fabrication.

## 5. Conclusions and Outlook

Herein, we overviewed systematic approaches to mimic natural polyphenols. Owing to the intrinsic adhesiveness and crosslinking ability of polyphenols, researchers have adopted the underlying chemistry to develop various polyphenolic materials. This has been applied to various applications, particularly biomedical applications. Considering the versatility of the materials and their numerous potential applications, we expect that the development of polyphenolic materials will continue. Future studies can address the following current limitations.

### 5.1. Current Limitations

Only an extremely limited number of polyphenolic materials have been commercialized despite a decade of research. This is believed to be due to the following reasons: (1) the oxidation kinetics of the polyphenolic materials are hard to control under ambient conditions, and the (2) chemical structure of the polyphenolic materials is not well-defined, which limits the prediction of side effects in an in vivo environment. We outline the practical difficulties related to the aforementioned problems regarding the practical realization of polyphenolic materials.

Considering that ambient air contains ~20% of oxygen, polyphenolic materials should gradually be oxidized, which may significantly affect the performance of the product, particularly for the wet-adhesive material. The packaging technique where oxygen is completely blocked from the external environment is essential in this case. Representative polyphenolic commercial products always arrive in a specialized packing system; hemostat (InnoSEAL™) developed by InnoTherapy Inc. (S. Korea, Seoul) and hair-dyeing shampoo (Black Change Complex™) developed by MODA MODA Inc. (S. Korea, Seoul) are typical examples. This is a limitation regarding the commercialization of polyphenolic materials.

Major phenolic derivatives such as catechols and gallols exhibit a quinone structure, which induces oxidative crosslinking. Unfortunately, the quinone undergoes complex conjugation. A significant amount of effort has been dedicated to defining the chemical structures of polyphenolic materials after oxidation [103]; however, until recently, the exact structure was undefined. Owing to this uncertainty, the ministries for food and drug safety are generally skeptical about using these materials, particularly for application to internal organs. Only a few cases in which backbone materials (polysaccharides) are the main materials and catechol/gallol moieties are substituted at less than 5% were approved by the U.S. Food and Drug Administration.

### 5.2. Suggestions

To overcome these aforementioned issues, basic building blocks that result in predictable chemical structures need to be developed. Ideally, the oxidative crosslinking behavior of these materials should be precisely controlled with a better understanding of the underlying mechanisms. In addition, fundamental studies on the biological responses of polyphenolic materials depending on their chemical structures, oxidation states, and external conditions should be deeply investigated. We believe that these efforts will enable the systematic design of polyphenol-based functional materials and accelerate the realization of practical applications of polyphenolic materials.

## Figures and Tables

**Table 1:** Types of backbone materials for polyphenolic functional materials.

Type			References
Synthetic Polymers	Polyethyleneimine (PEI)		[25,26]
	Polyethylene glycol (PEG)		[27,28]
	Polyethylene		[29]
	Polyester		[30,31]
	Epoxy		[32,33]
	Polypropylene		[34,35]
	Polytetrafluoroethylene		[36,37]
	Polystyrene		[38,39]
Protein-based Materials	Peptides		[40,41]
	Peptoids		[42,43]
	Extracellular matrix (ECM)		[44]
	Recombinant polypeptides	Mussel adhesive proteins (MAPs)	[45,46]
		Elastin-like polypeptides (ELPs)	[47,48]
Polysaccharides	Chitosan		[49,50]
	Hyaluronic Acid		[51,52,53]
	Alginate		[54,55]
	Collagen		[56]
	Cellulose		[57]
None	Polydopamine (pDA)		[58,59]
	Polynorepinephrine (pNE)		[60,61]
	Polytannic acid		[62,63]
	Pyrocatechol violet		[64]
	Polycatechin		[65,66]

**Table 2:** Backbone-dependent properties of polyphenolic materials (order indication: I > II > III).

	Synthetic Polymers	Protein-Based Materials	Polysaccharides	None
Biocompatibility	III	I	II	II
Accessibility	I	III	II	I
Degradation	III	I	II	III
Mechanical stability	I	III	II	II
